# Plasma Peptide Concentrations and Peptide-Reactive Immunoglobulins in Patients with Eating Disorders at Inclusion in the French EDILS Cohort (Eating Disorders Inventory and Longitudinal Survey)

**DOI:** 10.3390/nu12020522

**Published:** 2020-02-18

**Authors:** Marie Galmiche, Nicolas Lucas, Pierre Déchelotte, Camille Deroissart, Marie-Anne Le Solliec, Julie Rondeaux, Saida Azhar, Sébastien Grigioni, Guillaume Colange, Julie Delay, Najate Achamrah, Vanessa Folope, Liliana Belmonte, Adèle Lamarre, Agnès Rimbert, Tiphaine Saillard, André Petit, Muriel Quillard, Moise Coeffier, André Gillibert, Grégory Lambert, Romain Legrand, Marie-Pierre Tavolacci

**Affiliations:** 1 Inserm UMR1073, 76000 Rouen, France; marie.galmiche3@gmail.com (M.G.); sebastien.grigioni@chu-rouen.fr (S.G.); Najate.Achamrah@chu-rouen.fr (N.A.); vanessa.folope@chu-rouen.fr (V.F.); lilibelmonte3@hotmail.com (L.B.); agnes.rimbert@chu-rouen.fr (A.R.); Andre.Petit@chu-rouen.fr (A.P.); Moise.Coeffier@chu-rouen.fr (M.C.); MP.Tavolacci@chu-rouen.fr (M.-P.T.); 2 TargEDys SA, 91160 Longjumeau, France; lucasnicolas@hotmail.fr (N.L.); camillederoissart@gmail.com (C.D.); maleso@msn.com (M.-A.L.S.); julie.rdx@gmail.com (J.R.); saida-az@hotmail.fr (S.A.); glambert@targedys.com (G.L.); legrandromain@hotmail.fr (R.L.); 3 Institute for Research and Innovation in Biomedicine (IRIB), University of Rouen, 76000 Rouen, France; 4 Nutrition unit, University Hospital of Rouen, 76000 Rouen, France; guillaume.colange@chu-rouen.fr (G.C.); julie.delay@chu-rouen.fr (J.D.); Adele.Lamarre@chu-rouen.fr (A.L.); saillard.tiphaine@gmail.com (T.S.); 5 CIC-CRB 1404 INSERM, University Hospital of Rouen, 76000 Rouen, France; Muriel.Quillard@chu-rouen.fr; 6 Department of Biostatistics, Rouen University Hospital, F 76000 Rouen, France; Andre.Gillibert@chu-rouen.fr

**Keywords:** eating disorder, peptides, immunoglobulins, human, plasma

## Abstract

Eating disorders (EDs) are increasingly frequent. Their pathophysiology involves disturbance of peptide signaling and the microbiota–gut–brain axis. This study analyzed peptides and corresponding immunoglobulin (Ig) concentrations in groups of ED. In 120 patients with restrictive (R), bulimic (B), and compulsive (C) ED, the plasma concentrations of leptin, glucagon-like peptide-1 (GLP-1), peptide YY (PYY), and insulin were analyzed by Milliplex and those of acyl ghrelin (AG), des-acyl ghrelin (DAG), and α-melanocyte-stimulating hormone (α-MSH) by ELISA kits. Immunoglobulin G (in response to an antigen) concentrations were analyzed by ELISA, and their affinity for the respective peptide was measured by surface plasmon resonance. The concentrations of leptin, insulin, GLP-1, and PYY were higher in C patients than in R patients. On the contrary, α-MSH, DAG, and AG concentrations were higher in R than in C patients. After adjustment for body mass index (BMI), differences among peptide concentrations were no longer different. No difference in the concentrations of the IgG was found, but the IgG concentrations were correlated with each other. Although differences of peptide concentrations exist among ED subtypes, they may be due to differences in BMI. Changes in the concentration and/or affinity of several anti-peptide IgG may contribute to the physiopathology of ED or may be related to fat mass.

## 1. Introduction

Eating disorders (EDs) are a public health issue, characterized by important disturbances of food behavior, body image, and corpulence [1]. EDs are ubiquitous and the lifetime prevalence for all EDs ranges from 3.3% to 18.6% for women and from 0.8% to 6.5% for men [2]. As an alternative to the DSM-5 precise ED definition (standard classification), some authors have proposed to classify ED according to broad categories featuring the main symptom [3]. This classification based on pragmatic clinical approach describes three broad categories: Restrictive (R), bulimic (B), and compulsive (C) disorders. 

Eating disorders are multifactorial diseases depending on the combination of genetic [4] and psychological factors [5,6], in close interaction with family, environmental, socio-cultural, and homeostatic factors [7,8]. The mechanisms of homeostatic regulation of eating behavior are regulated by numerous signaling pathways integrated in the hypothalamus [9]. Among these, peptides play a key role as anorectic or orexigenic factors [10]. Several studies display alterations of these peptides’ plasma concentrations during ED, which could be indicative of or contribute to the onset and/or maintenance of the pathology. 

The main anorexigenic peptides are leptin, insulin, peptide YY (PYY), glucagon-like peptide-1 (GLP-1), and α-melanocyte-stimulating hormone (α-MSH). At this time, only one orexigenic hormone has been demonstrated: Acyl ghrelin. This hormone comes from the non-octanoylated form, des-acyl ghrelin, which would have an anorectic role [11]. 

These changes in peptide concentrations and/or effects could be related to dysbiosis of gut microbiota, characterized by a change in the number or nature of bacteria present in the gut [12]. 

Moreover, the gut microbiota is also known to have an impact on the immune system and especially its maturation [13]. The dysbiosis of the gut microbiota during ED could impact the concentration of the circulating peptides but also modulate immunity by leading to an inappropriate process and thus by having an impact on immunoglobulin (Ig) concentrations and their affinity [14,15,16]. In germ-free mice, a decrease in Ig concentrations associated with an alteration of intestinal functions was observed [17]. 

The involvement of autoantibodies on the hypothalamic system and thus on the regulation of food intake of AN and BN patients was already suggested in 2002. Indeed, anti-α-melanocyte-stimulating hormone and anti-adrenocorticotropic hormone (anti-ACTH) antibodies from these patients were able to bind to melanocortins and corticotrophin-containing neurons from the hypothalamus of rats. These autoantibodies anti-α-MSH and anti-ACTH could be produced as a result of concomitant activation of the hypothalamic-pituitary-adrenal axis and the immune system. In fact, the activation of the hypothalamic-pituitary-adrenal axis is a characteristic feature of AN [18]. 

The different functional properties of these Ig, such as affinity and plasma concentration, could either reduce or reinforce the biological activity of the peptide on eating behavior [19,20,21]. In fact, a change in the affinity of IgG and IgM (in response to first contact with an antigen) against α-MSH within the adaptive response to food deprivation was observed in rats [22]. The production of α-MSH-directed Ig could be influenced by the combination of stress, food restriction, or altered intestinal permeability [23]. Finally, we previously reported that some proteins, such as caseinolytic peptidase B (ClpB), may share some effect with α-MSH by molecular mimicry [20,21]. Indeed, a part of the ClpB protein produced by Enterobacteriaceae, such as *E. coli* or *H. alvei*, presents a molecular mimicry with α-MSH, which can reduce food intake in rodents [19,20,21]. In a preliminary study, plasma ClpB concentrations were increased in patients with ED as compared to controls, but without a difference among AN, BN, and BED [21].

To allow a comprehensive assessment of clinical and biological features in patients with well-characterized ED, the EDILS prospective cohort (Eating Disorders Inventory and Longitudinal Survey) was launched at Rouen University Hospital. Based on the analysis of the first 120 patients included in this cohort, the main objective of the present study was to measure the different peptides involved in the regulation of eating behavior (leptin, insulin, GLP-1, PYY, α-MSH, and acyl and des-acyl ghrelin) and their corresponding Ig in three groups of patients with ED of the restrictive (R), bulimic (B), or compulsive (C) types. This study also allowed study of the different associations between biological data but also between clinical co-morbidities and biological parameters. 

## 2. Materials and Methods

### 2.1. Study Design: EDILS Cohort

The EDILS cohort with biocollection was launched in 2015 after approval by the French national committee “Informatique et liberté” (N° CNIL: 1787487) and the Ethics Committee (N° CPP/CE 002/2014). All patients aged 18 or older referring to the Nutrition Department for the first consultation for a non-treated ED were potentially considered for inclusion. During the first consultation, the physician established the ED diagnostic according to the DSM-5 classification. After agreeing to participate and having given written consent, patients were invited to fill in the first questionnaire and received a stool collection kit. The questionnaire collected at patients’ inclusion enabled description of their clinical features, including anthropometric, sociodemographic, and addiction risk data. In addition, standardized EDI-2, BSQ, and HAD questionnaires were filled in. The EDI-2 has been validated to determine personality traits and comorbidities frequently associated in patients with ED [24]. The Body Shape Questionnaire (BSQ) is a standardized questionnaire assessing patients’ bodily concerns [25]. Finally, the HAD questionnaire was used to evaluate anxiety and depression [26]. In order to determine significant differences according to EDI-2 and BSQ, the cut-off corresponds to the median of the scores. For the HAD sub-item, the scores higher than the cut-off (11) mean that patients have certain anxiety/depression and vice versa. 

All data used in this article are from the first self-questionnaire provided to the 120 patients enrolled between April 2015 and January 2018.

As part of their usual care, patients came back to the hospital within a few weeks for a comprehensive evaluation on a morning day hospital basis. Patient plasma was collected between 8 and 11 in the morning after an overnight fast. In addition to routine blood samples, aprotinin- and heparin-containing tubes were taken and shortly after centrifuged for 15 min at 4 °C at 3500 rpm; 1–3 aliquots of 600 µL per each tube were prepared and frozen at −80 °C until analyses. 

Although the DSM-5 is recognized as the gold standard for the diagnosis of ED, the difference among typical and atypical ED in this classification is mainly related to the degree of severity while the clinical features are essentially similar. In addition, it has been proposed that broad categories of ED (restrictive, bulimic, and compulsive) are useful for the clinical approach of ED [2,27]. Finally, in the biological analysis of the present study, there were no statistically significant differences between results from patients with typical versus atypical ED, the only exception being for PYY. Therefore, in order to combine biological and clinical profiles, we pooled the patients included in this study in 3 broad categories of restrictive (R), bulimic (B), and compulsive (C) ED. The R category includes AN, restrictive food intake disorder, and atypical AN; B category includes BN or atypical BN of low frequency or duration; and the C group includes BED, BED of low frequency or duration, and night eating syndrome. “Restrictive” patients are characterized by a Body Mass Index (BMI) lower than normal according to the WHO [BMI <18.5]. “Bulimic” patients are normal weight or overweight [18.5 < BMI < 30]. On the contrary, “compulsive” patients have obesity and therefore a BMI greater than 30.

Clinical and biological data will be presented in the following sections according to these 3 broad groups, except for PYY concentrations for which data will be presented separately according to typical and atypical ED.

### 2.2. Peptide Concentrations

GLP-1(7-36), PYY (3-36), leptin, insulin, α-MSH, and des-acyl ghrelin (DAG) were the anorexigenic peptide concentrations measured; acyl ghrelin (AG) was the orexigenic peptides measured. 

Plasma concentrations of AG, DAG, and α-MSH were measured using an enzyme-linked immunosorbent assay kit (ELISA kit) and following the supplier instructions. The coefficients of variation for the measurement of peptide concentrations were less than 10%.

Plasma concentrations of leptin, insulin, GLP-1, and PYY were measured using a Milliplex Map Kit with the associated protocol (Merck Millipore, Darmstadt, Germany). The coefficient of variation for these 2 peptide concentrations was less than 15%. The choice of the form of the peptide studied was also chosen according to the literature. PYY (3-36) is more potent than PYY (1-36) in inhibiting gastric emptying [28]. GLP-1(7-36) analysis was chosen because it is the major circulating bioactive species in humans [29]. 

### 2.3. IgG Concentrations

Plasma concentrations of IgG anti-acyl ghrelin, des-acyl ghrelin, PYY, GLP-1, insulin, and leptin were measured using an enzyme-linked immunosorbent assay technique (ELISA) according to an already published protocol [30]. The total and free IgG concentration for each peptide was measured. The coefficients of variation of the IgG concentrations were less than 10%.

### 2.4. Affinity Measurements

First, the IgG was purified with the MelonGel^®^ Purification kit (LifeTechnologies, Carlsbad, CA, USA) and according to supplier instructions. After purification, the IgG concentration of each sample was measured using Nanodrop 2000 C (ThermoFisher Scientific, Waltham, MA, USA) with HBS-EP buffer as the blank. 

The affinity of patients’ IgG for each peptides of interest were determined by surface plasmon resonance (SPR) with BIAcore T200 (GE Healthcare, Velizy Villacoublay, France). For the coating, peptides were diluted at 0.5 mg/mL in 10 mM sodium acetate buffer (pH 5.0) and 250RU of peptides were covalently coated on 3 cells of the CM5 sensor chip (GE Healthcare), using an amine coupled kit (GE Healthcare). Affinity kinetic analysis was performed using a single-cycle method with five serial dilutions at ½ of each IgG sample from 840 mM to 52.5 nM. Here, 60 µL of each sample dilution were injected at 30 µL/min followed by 5 min of dissociation. Finally, the binding surface was regenerated with 50 mM NaOH resulting in the return of the sensorgram baseline. The affinity kinetic data were analyzed with the BioEvaluation 4.1.1 program (GE Healthcare) and kinetics curves were fitted using Langmuir’s 1:1 model after correction with the reference cell. For affinity measurements, the coefficients of variation are less than 5%.

### 2.5. Statistical Analysis 

Data were analyzed in the Xlstat and R statistical software (version 3.5.0, The R Foundation for Statistical Analysis). Comparison of clinical data among ED subtypes groups was performed by the Kruskal–Wallis test, but the comparison of the sex ratio was performed by Fisher’s exact test.

All biological data were rank transformed before multivariable analysis to be consistent with rank tests; then, ranks were divided by the number of observations and multiplied by 100 in order to express these variables as empirical percentiles of the overall distribution (three ED groups pooled). Unadjusted, age- and sex-adjusted, then age-, sex-, and BMI-adjusted general linear models were then estimated on the transformed variables (percentiles). The small number of patients in the bulimic group did not allow a reliable statistical interpretation; the results were thus presented just as descriptive data. So, the C group was compared to the R group.

The correlogram (graphical representation of a correlation matrix) was made using the Spearman’s correlation coefficient.

Three principal component analyses (PCAs) were performed after percentiles transformation for: (1) All IgG quantifications, (2) all IgG affinities, and (3) all peptides measures. These PCAs were designed to perform a dimensional reduction of the data set and their performances were analyzed by scree plots, showing eigenvalues of the data set associated to the eigenvalues of 20 simulated PCAs in a dataset of the same size but with no correlation between any biological variable.

Twenty-three fully adjusted (adjusted on age, sex, and BMI) comparisons of percentiles of raw biological variables among ED subtypes were performed with a Bonferroni multiple testing procedure keeping a family-wise error rate (FWER) equal to 5%. The two comparisons of the first component of PCAs of IgG concentrations and IgG affinities were performed with a second Bonferroni FWER equal to 5%. Ninety-two age-, sex-, ED subtype-, and BMI-adjusted tests were performed in general linear models to assess the correlations between four clinical variables (BSQ, EDI-2, anxiety subscale of HAD, depression subscale of HAD) and biological variables; a third FWER at 5% was applied for these comparisons. Eight more tests were performed with the first components of PCAs of IgG concentrations and IgG affinities; a fourth FWER at 5% was applied for these comparisons. Comparisons of clinical variables among ED subtypes were performed at the 5% significance threshold without multiple testing corrections.

## 3. Results

### 3.1. Characteristics of the Patients

As described in the methods section, the 120 patients included were pooled in 3 broad categories. The R group included 35 patients (17 with typical AN, 5 patients with atypical AN, and 13 patients with restrictive eating disorder). The B group included 12 patients (7 with typical BN and 5 with atypical BN). Finally, the C group included 67 patients (38 with typical BED, 29 with atypical BED and 6 with night eating syndrome). 

The population was composed of mainly female patients, with only 14% of men (Table 1). As expected, the mean BMI was significantly lower in the R than in the B and C group (16.4 kg/m² vs. 23.2 kg/m² and 38.1 kg/m²; *p* < 0.001). Patients from the R group were significantly younger than those from the C group but not from the B group (29.4 vs. 39.4 years and 36.0; *p* < 0.001 and *p* = 0.13).

No significant differences in the EDI-2 total score among the three ED forms were found. However, the sub-items of EDI-2 body dissatisfaction and impulse regulation were significantly higher in C patients than R and B patients. As expected, bulimia was significantly higher in B groups compared to others (Table 1). 

The total BSQ and each sub item were significantly higher in the C group than in the R and B groups (Table 1). 

### 3.2. Peptide Concentrations

**Figure 1:**
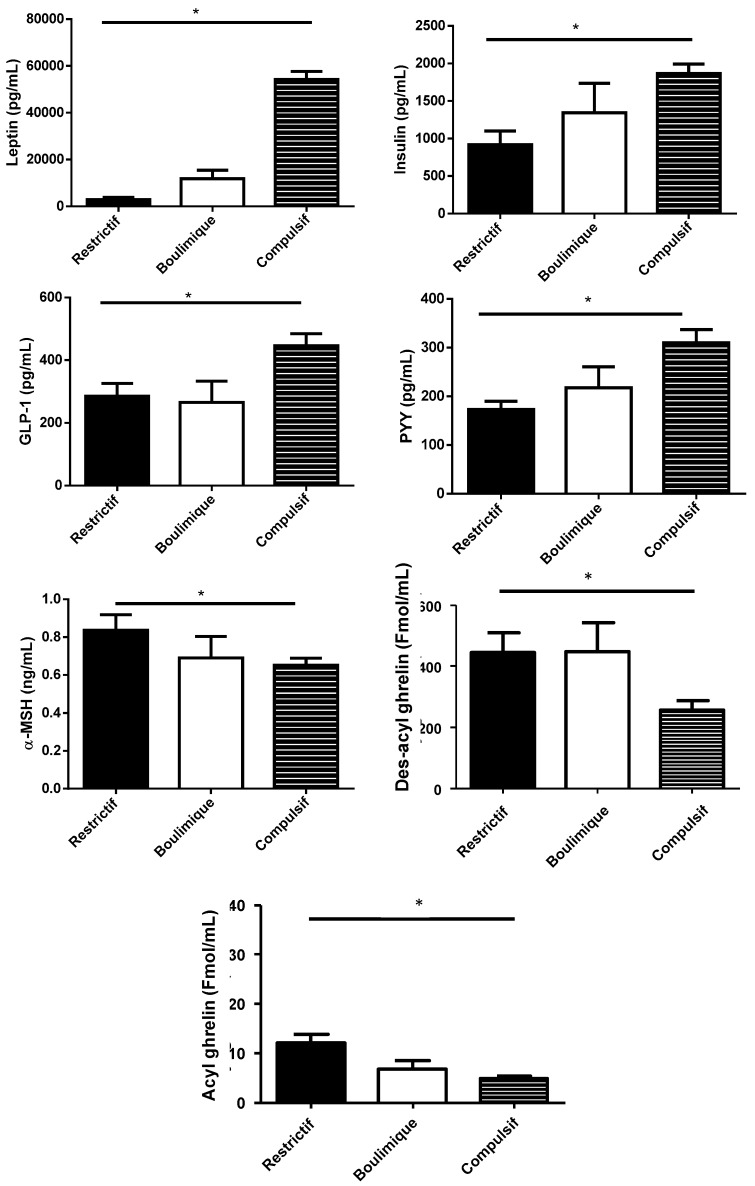
Plasma concentration of peptides associated with food intake according to broad categories of ED. * Significance after adjustment on age and sex. The standard deviation is represented by the error bar.

Plasma concentrations of the anorexigenic hormones leptin, insulin, GLP-1, and PYY were higher in C compared to R patients (Figure 1). In contrast, α-MSH, des-acyl ghrelin, and acyl-ghrelin concentrations were higher in R compared to C patients (Figure 1). Statistical comparisons for non-adjusted and adjusted data are presented in Table 2. Adjustment on age and sex did not notably change the level of statistical significance. After additional adjustment on BMI, peptide concentrations were no longer statistically different.

### 3.3. Immunoglobulin Concentrations and Affinity

The concentrations of free and bound IgG were measured. Since the level of free IgG is much lower than the concentration of bound IgG (four times, except for anti-ClpB IgG) and since no significant difference was found for free IgG among ED groups, the results were presented as total IgG. 

**Figure 2:**
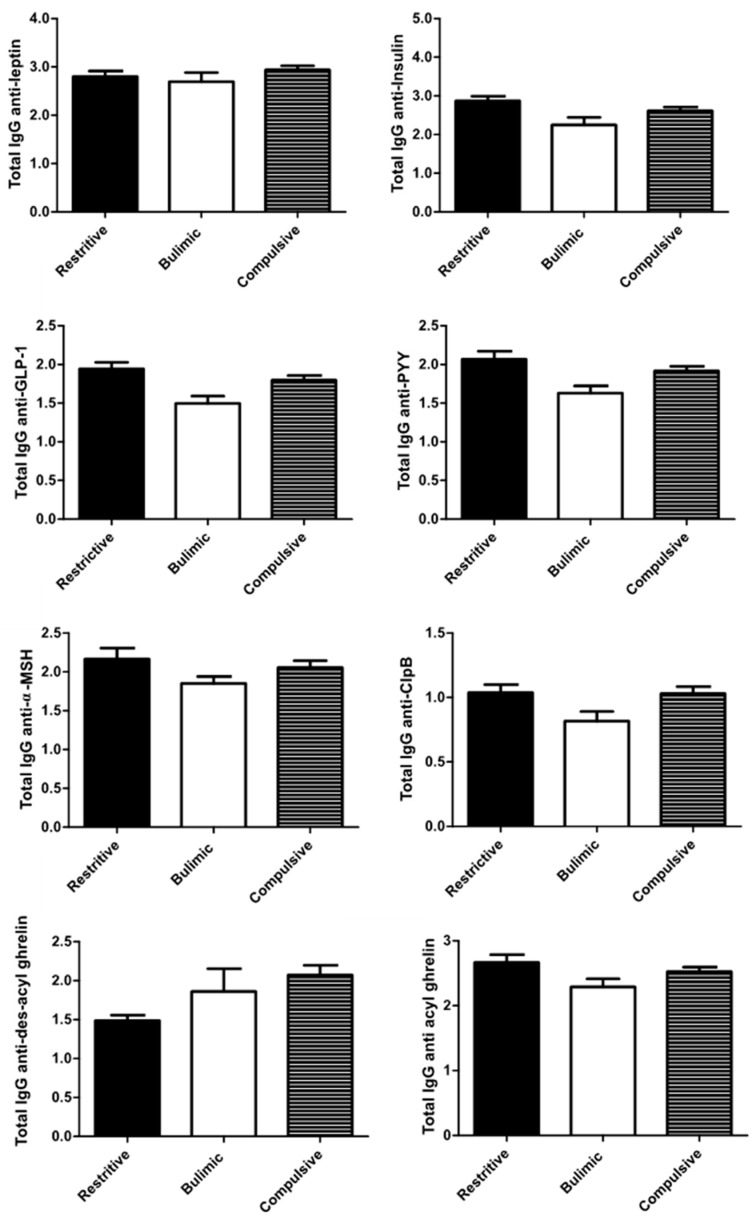
Total plasma IgG against peptides associated with food intake according to broad categories of ED. The standard deviation is represented by the error bar.

Except for the anti-DAG IgG, all IgG profiles are broadly similar, i.e., a lower rate in bulimic patients compared to the other two groups (Figure 2).

With or without adjustment, there was no significant difference between the concentrations of different IgGs among C and R patients (Table 3).

**Figure 3:**
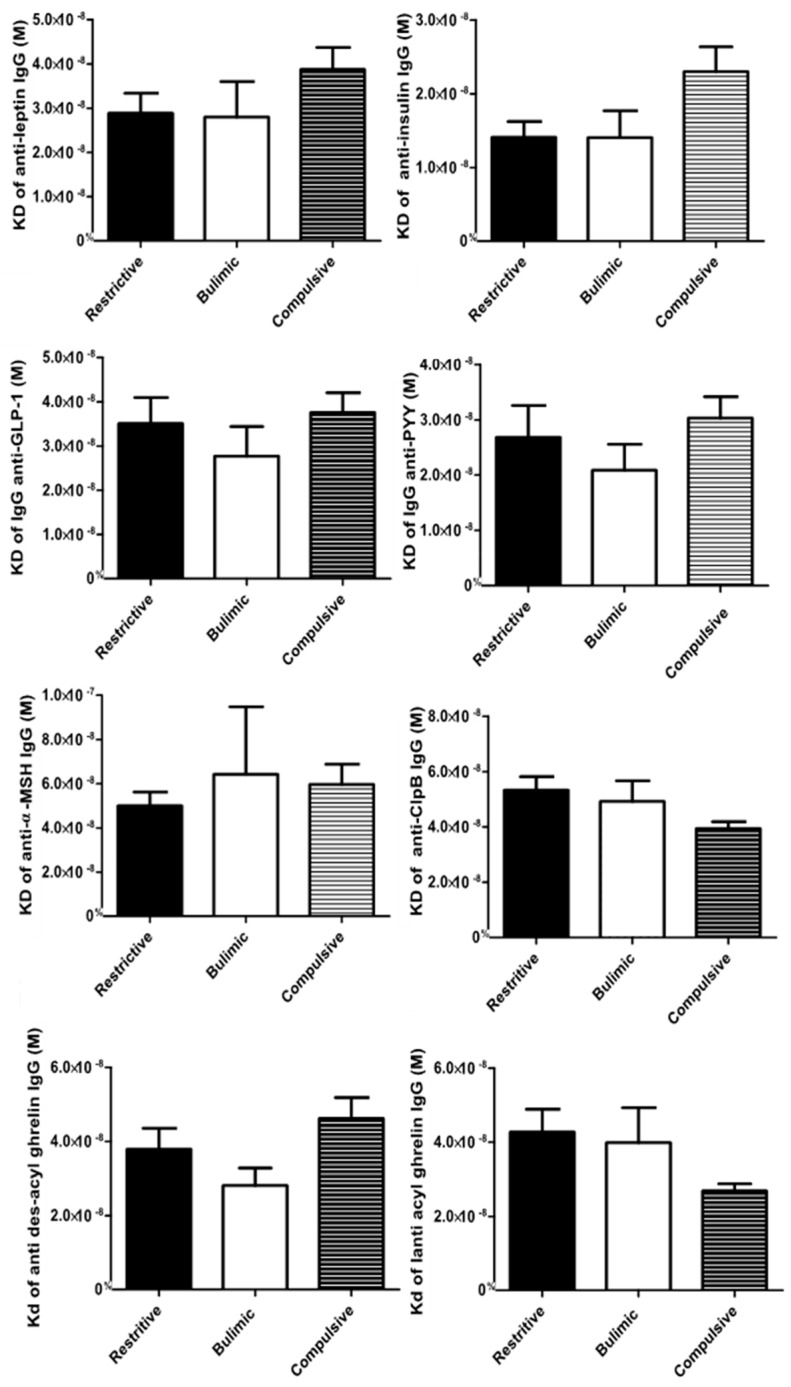
Affinity of plasma IgG against peptides associated with food intake according to broad categories of ED. The standard deviation is represented by the error bar.

The affinity profiles among the three broad categories of TCA were different according to IgG (Figure 3). Before adjustment, the affinity of anti-acyl ghrelin and anti-ClpB IgGs was significantly lower in the R group compared to the C group (Table 3). Adjustment on age and sex did not change the results, whereas after adjustment on the BMI, the affinity difference among the C and R group was no longer significant.

### 3.4. Association between Biological Data/Principal Component Analyses (PCAs)

**Figure 4:**
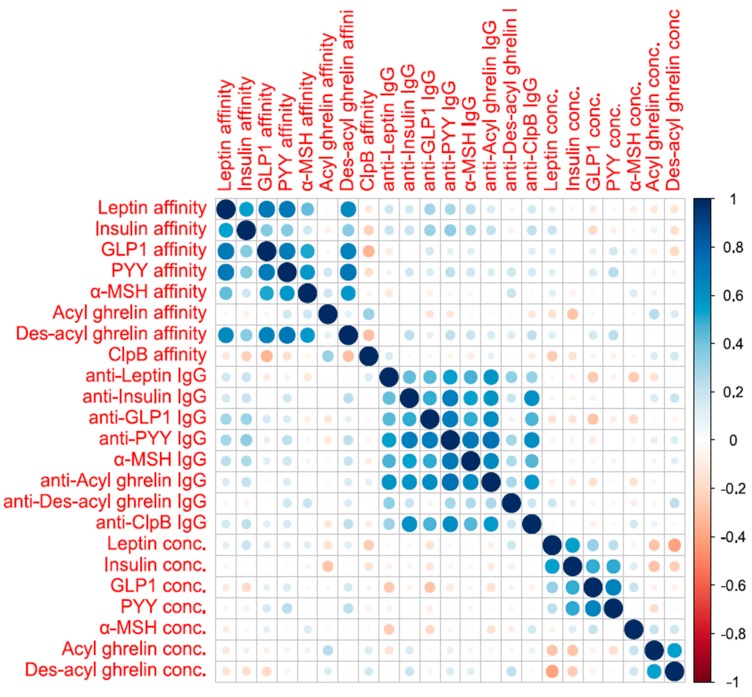
Correlogram representing all the correlations between the studied biological variables.

The correlogram (Figure 4) showed that IgG concentrations had weak to medium correlations with each other. The same was observed for the affinities of various IgG. On the other hand, the affinities and concentrations of the IgG were not correlated. Finally, peptide concentrations had weak positive and negative correlations with each other (Figure 4).

The first principal component of the principal component analysis (PCA) of IgG concentrations explained 56% of the variance of all IgG concentrations, which was considered acceptable to use the first principal component as a new variable for analysis. Variance explained by the first principal component of IgG affinities explained 48% of the variance, but the explained variance for peptide concentrations (38%) was considered as too low for analysis as the variance explained by chance assuming no correlation between concentrations was 20%.

Therefore, the PCAs allowed construction of two variables respectively representing the IgG concentration and their affinity.

The first principal component of the IgG concentration was expressed as the following linear combination:

0.36 × Leptin IgG conc + 0.38 × Insulin IgG conc + 0.37 × GLP1 IgG conc + 0.45 × PYY IgG conc + 0.40 × α-MSH IgG conc + 0.43 × Acylghrelin IgG conc + 0.19 × Desacylghrelin IgG conc.

The first principal component of the IgG affinity was expressed as the following linear combination:

0.36 × Leptin IgG KD + 0.38 × Insulin IgG KD + 0.37 × GLP1 IgG KD + 0.45 × PYY IgG KD + 0.40 × α-MSHIgG KD + 0.43 × Acylghrelin IgG KD + 0.19 × Desacylghrelin IgG KD.

The first component of the IgG concentrations was not significantly increased in R patients compared to C patients, without adjustment (+4.3, 95% CI: −7.2 to +15.8, *p* = 0.92 on the percentile scale) and with adjustment on age, sex, and BMI (−6.8, 95% CI: −30.7 to +17.0, *p* = 1.00). The first component of IgG affinities was not significantly decreased in R patients compared to C patients, without adjustment (−5.9, 95% CI: −17.6 to 5.8, *p* = 0.64 on the percentile scale) and with adjustment on age, sex, and BMI (−5.7, 95% CI: −30.4 to +19, *p* = 1.00). 

### 3.5. Association between Clinical and Biological Data

Unadjusted correlations of clinical variables to biological variables showed a significant correlation between leptin concentration and BSQ (*p* = 0.0007 after multiple testing correction for 92 tests). An increase in 100 percentiles of BSQ was associated to an increase of 39.6 (95% CI: 22.8 to 56.3) percentiles of leptin without adjustment. After adjustment on age, sex, and ED subtype, no association was significant. After further adjustment on BMI, no association was significant (all *p*-values > 0.10)

The analysis of correlations between the clinical variables and the first components of IgG concentrations and affinities PCAs showed no significant correlation without adjustment or with adjustment.

## 4. Discussions

**Figure 5:**
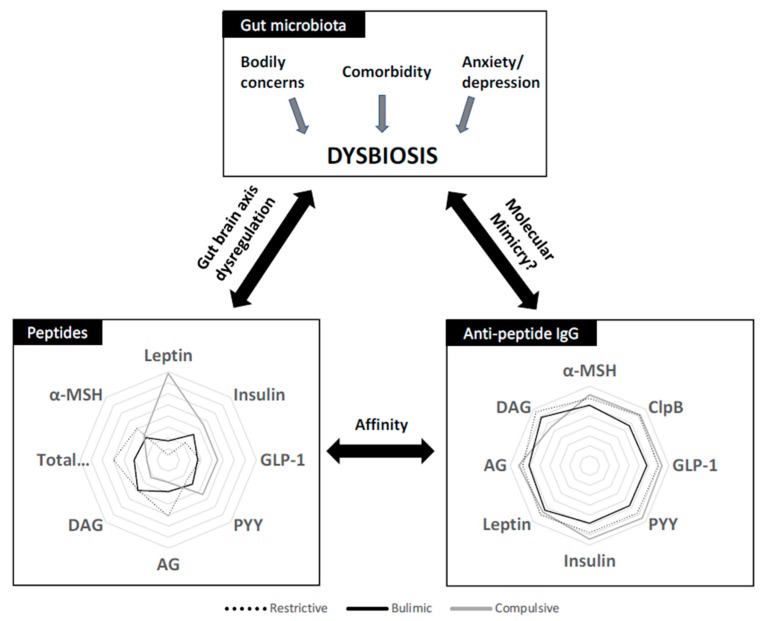
Interaction between gut microbiota, peptides, and immunoglobulin G concentrations in broad categories of ED (restrictive, bulimic, and compulsive).

This study provides for the first time a comprehensive pattern of peptides and their plasma IgG concentrations in three groups of untreated patients with well-characterized ED (Figure 5). In terms of prevalence, the population included is fairly representative of the usual clinical population, with R, B, and C patients accounting for 29%, 10%, and 61% of the recruitment. The C disorders prevail over R and B in accordance with previous prevalence studies, and this case mix is close to the estimation of the prevalence of ED in France [31]. Most interestingly, all these patients were included before the initiation of care in the Rouen University Hospital, and may thus be considered as “naive” patients, which prevents from bias related to previous therapeutic interventions. However, a few patients in each group did receive some non-specific symptomatic treatments, such as anxiolytics (mainly benzodiazepins) or spasmolytics (e.g., phloroglucinol) for abdominal discomfort, which is commonly associated to ED.

The mean interval between the onset of disease and the first specialized consultation was 5, 7, and 10 years for the R, B, and C group, respectively, and patients with C were older than in patients of other groups. This is a common finding that the severity and the earlier onset in the life of R and B disorders lead to a referral for care at a younger age and conversely for C patients [32]. 

In this study, patient plasma was collected in the morning in the fasting state. In addition, a statistical test analyzing the results of the plasma analyses according to the time of collection (between 8 and 11 a.m.) was performed and showed no significant difference.

In humans, obesity is associated with an increase in leptin concentrations, suggesting that leptin signaling is impaired. In our cohort, the leptin concentration was higher in C patients compared to R ones. As expected, this difference was no longer significant after adjustment on BMI and it is impossible to determine the influence of BMI regardless of the diagnostic group. In fact, BMI is a clinical parameter that generally facilitates the diagnosis (and reflects the patient’s ED form). Resistance to leptin in C patients could thus be induced by weight gain and therefore by hyperphagia. This may be related to a change in its transport at the blood–brain barrier, leading to ongoing ingestion of rewarding foods, and reinforcing the addictive hyperphagic behaviors [33,34]. 

GLP-1 and PYY are secreted by the endocrine cells of the proximal small intestine and distal small intestine during the passage of nutrients [35,36,37]. These two peptides induce delayed gastric emptying and satiety signaling [38]. The concentrations of both were increased in the C group compared to the R group, suggesting resistance to these peptides in these patients, as previously reported in obese and binge-eating patients [39]. Binge eating is characterized by massive ingestion of high-fat and high-sugar food, which may stimulate an increased release of PYY and GLP-1 by endocrine cells either directly, by a nutrient effect, or indirectly [21,40] with the induction of dysbiosis and the overstimulation of endocrine cells by microbiota-derived signals.

Increase of plasma insulin in response to food intake, mostly glucose and some amino acids, also contributes to the signaling of satiety acting at the hypothalamic level [41], as evidenced by the reduction of food intake after an intracerebroventricular injection of insulin. The increased insulin plasma concentrations observed in C patients in the present study could result from two additive mechanisms: The direct endocrine response to massive carbohydrate ingestion and the reduced central clearance of insulin resulting from downregulation of insulin receptors’ expression in the hypothalamus [42] in response to repeated binging [43]. 

Only few studies have reported plasma concentrations of α-MSH during ED, limiting the knowledge on these biological changes in AN [44,45]. Circulating α-MSH may be from peripheral or central origin. One study showed a decrease in the plasma α-MSH concentration in AN compared to controls all along the day [44] while another study reported no significant difference among AN and controls [45]. In the physiological situation, α-MSH is secreted by the activation of POMC neurons in the arcuate nucleus by some signals, such as leptin, an increased leptin concentration signaling replenishment of energy stores leads to increased POMC expression, and α-MSH release, which finally reduces food intake [46]. For the first time, our results show a comparison of plasma α-MSH concentrations among the three main groups of ED and no significant difference was found among ED. In rats, repeated exposure to mild stress increased the concentrations and affinity of α-MSH-reactive IgG that could modify α-MSH signaling [22]. In another study, anti α-MSH IgG concentrations decreased during gut mucositis in rats, a model associated with prolonged anorexia [47]. Different types of stress may have different effects on the anti-MSH IgG concentration. In the present study, we observed no significant difference in the anti-α-MSH IgG concentrations and their affinity among the three groups. This is in agreement with another report from our group comparing anti-MSH IgG concentrations among AN, BN, and BED (DSM-IV) patients [48]. It is worth discussing if some bacterial signaling molecules may contribute to the induction of satiety in a way that is similar to endogenous α-MSH. Indeed, a part of the ClpB protein produced by Enterobacteriaceae, such as *E. coli* or *H. alvei*, presents a molecular mimicry with α-MSH, which can reduce food intake in rodents. In a preliminary study, plasma ClpB concentrations were increased in patients with ED as compared to controls, but without differences among AN, BN, and BED [21]. In this same study, no significant difference was observed for anti-ClpB Ig among groups. In the present study, in well-defined groups of naïve patients from a single center, we observed increased anti-ClpB Ig in R and C patients as compared to B. In addition, the affinity of anti-ClpB Ig was increased in C as compared to R patients. This may contribute to blunting the satiating effect of ClpB in C patients by limiting the binding of this peptide on its receptor, and also limit the effect of α-MSH by cross-reaction between this peptide and anti-ClpB Ig.

Several studies have reported increased total ghrelin concentrations during AN while it is reduced during BED. In our study, we confirmed the increased total ghrelin in R patients as compared to B and C groups. Interestingly, both AG and DAG concentrations were increased in R patients, and DAG accounted for 96% of total ghrelin, which confirms a previous study where AG concentration represented only 2–5% of total ghrelin in rodents and 10% in humans. DAG may act both as a direct inhibitor of AG and also by decreasing plasma AG concentration. The acylation of ghrelin may be modulated by the diet [49,50], especially by lipid intake; indeed, a high consumption of medium-chain fatty acids increased the AG concentration [51]. Thus, during restrictive ED, the limitation of energy intake may elicit increased ghrelin secretion and may simultaneously impair the activity of ghrelin O acyl transferase (GOAT), resulting in an increased proportion of DAG, and finally to a resistance to the effect of AG perpetuating reduced food intake. Some previous studies in the literature have shown that modulation of the signaling of ghrelin would be possible through Ig. In a former study in a small group of patients with AN, anti-AG IgG was decreased as compared to healthy volunteers while anti-DAG IgG was not different [52]. Another study reported similar results [53]; the affinity of anti-AG IgG was decreased in AN patients compared to obese [53,54]. Increased affinity of anti-AG IgG in obese patients was associated with in vitro protection of ghrelin and potentiation of its orexigenic effect after passive transfer in mice [53]. In this study, the affinity of anti-AG IgG tented to be lower in the R than C group, which could limit AG protection and reduce its orexigenic effect. It looks unlikely that the anti-DAG IgG concentration influences the biological actions of DAG, since DAG is found mainly in the unbound form in AN as well as obese patients and in controls [53]. Thus, elevated DAG by itself, regardless of Ig, may play a key role in the perpetuation of reduced food intake.

No significant difference in the concentrations of the different IgGs against peptides on food intake was found. However, the correlogram underlines an association between the concentrations of the different IgGs (except for anti-DAG IgG), which may mean that IgG concentrations altogether may reflect the presence of an ED. For logistic and regulatory reasons, the inclusion of healthy volunteers in the EDILS study started after the inclusion of ED patients. The future comparison of a larger group of patients to healthy volunteers paired on age, sex, and BMI (in the case of B patients) will determine whether an increase or decrease in IgG concentrations is representative of the change in eating behavior or only modifications of body mass.

Indeed, all patients in the present study displayed high anxiety and depressive HAD scores, with a mean total HAD score of 19 as compared to the usual value of 8.4 in the general population [55]. Similarly, EDI-2 scores in our ED patients ranged between 73 and 86 while the usual score in the general population is around 33.5 [56]. Finally, the mean BSQ score in the C group (101) was higher than the mean of 74.5 reported in the general population [57]. Bodily dissatisfaction is tightly correlated with low self-esteem in women [58] and also promotes depressive behavior [59]. Thus, EDs are tightly associated with anxiety, depression, and bodily dissatisfaction, which are comorbidities well known to have an important link with intestinal dysbiosis, suggesting that the comorbidities associated with ED might be at the origin or the intricated consequences of dysbiosis altering peptide signaling directly or via the modulation of IgG [12,60]. 

Before closing, a limitation of our manuscript should be mentioned. In particular, in the present study, we evaluated the circulating levels of gut peptides in a single time-point. As demonstrated in medical literature [61,62,63], the altered secretion in eating disorders should be investigated dynamically with more time-points. Nevertheless, as specified in our protocol, the sampling conditions were strictly standardized: The patient’s blood sample was drawn at 08.00–11.00 a.m. after an overnight fast. In addition, we found no statistical association between circulating levels of each gut peptide and the exact time of blood sampling. 

A that stage, due to the limited number of patients in the different groups, these results should be considered as preliminary and generate hypotheses that need to be confirmed on a larger study population.

In conclusion, this study provides new information about orexigenic and anorexic peptides and corroborates some previous indication of peptide-altered signaling in different groups of ED. Figure 5 proposes an integrative view of our data on peptides and Ig in the different groups of ED studied. Although differences in the peptide concentrations exist among ED subtypes, they may be due to the differences in BMI. In some but not all instances, we can speculate that ED- or BMI-related peptide-resistance patterns are related to the modulation of the respective IgG concentration or affinity. Further studies are ongoing to establish whether intestinal dysbiosis is implicated in the modulation of the production by the microbiota of peptides or peptide mimetics regulating food intake and eliciting the intestinal or systemic production of antipeptide IgG. An ongoing recruitment of healthy volunteers in our service opens the perspective of a comparative analysis of these peptides and immunoglobulins with a larger population of ED patients over a wide range of body mass.

## Figures and Tables

**Table 1:** Characteristics of patients according to the form of ED (*n* = 120).

Characteristics	Restrictive	Bulimic	Compulsive	*p*-Value
Men/Women	2/33	1/11	14/59	0.98
**BMI** (kg/m^2^)	**16.4 ± 2**	**23.2 ± 6**	**38.1 ± 6.8**	**<0.001**
**Age** (Years)	**29.4 ± 11.2**	**36.0 ± 16.5**	**39.4 ± 12.3**	**<0.001**
EDI-2	77.5 ± 41.8	85.3 ± 29.2	90.4 ± 34.1	0.18
*Drive for thinness*	9.1 ± 6.9	12.1 ± 5.2	10.2 ± 5.1	0.28
*Bulimia*	**2.1 ± 4.4**	**8.8 ± 5.2**	**5.6 ± 5.9**	**<0.001**
*Body dissatisfaction*	**11.1 ± 7.0**	**13.9 ± 7.0**	**20.3 ± 7.4**	**<0.001**
*Ineffectiveness*	9.8 ± 7.3	8.5 ± 6.8	9.6 ± 7.4	0.92
*Perfectionism*	6.7 ± 4.6	7.0 ± 4.7	5.3 ± 4.1	0.23
*Interpersonal distrust*	5.7 ± 4.3	4.8 ± 3.5	5.0 ± 3.7	0.83
*Interoceptive awareness*	8.2 ± 6.8	9.3 ± 4.5	8.3 ± 6.6	0.59
*Maturity fears*	6.3 ± 5.1	4.0 ± 3.8	5.8 ± 4.3	0.26
*Asceticism*	5.3 ± 3.3	5.9 ± 2.9	5.5 ± 3.3	0.65
*Impulse Regulation*	**5.5 ± 6.2**	**5.8 ± 4.6**	**7.2 ± 4.6**	**0.04**
*Social Insecurity*	7.8 ± 3.4	5.1 ± 3.1	7.0 ± 4.0	0.10
**BSQ**	**74.6 ± 34.0**	**96.2 ± 28.9**	**101.4 ± 24.3**	**<0.01**
*Using laxatives and vomiting in order to reduce body dissatisfaction*	**5.9 ± 3.9**	**7.7 ± 3.1**	**5.3 ± 2.0**	**0.03**
*Unsuited cognitions and behaviors in order to control the weight*	**16.0 ± 7.1**	**21.5 ± 6.6**	**19.0 ± 4.7**	**0.01**
*Body dissatisfaction compared to the lower parts of the body*	**32.7 ± 16.9**	**43.1 ± 13.1**	**47.2 ± 12.2**	**<0.01**
*Social avoidance and shame of the exposure of the body*	**19.9 ± 8.2**	**23.9 ± 10.4**	**30.0 ± 8.6**	**<0.001**
HAD				
*Anxiety*	11.3 ± 4.6	12.3 ± 4.3	10.5 ± 4.6	0.17
*Proven anxiety (Score > 11)*	68%	81%	49%	
*Depression*	8.4 ± 4.2	7.9 ± 4.1	8.1 ± 4.3	0.79
*Proven depression (Score > 11)*	29%	72%	31%	

BMI: Body Mass Index; EDI-2: Eating Disorder Inventory; BSQ: Body Shape Questionnaire; HAD: Hospital anxiety and depressive scale; Mean ± SD. The significant differences are in bold.

**Table 2:** Linear models explaining peptide concentrations associated with food intake according to different adjustments.

Peptides Conc.	Restrictive vs. Compulsive * Unadjusted Models	*p* **	Restrictive vs. Compulsive * Adjusted Models 1 ^†^	*p* **	Restrictive vs. Compulsive * Adjusted Models 2 ^‡^	*p* **
Leptin	−51.3 [−58 to −44.5]	<0.0001	−51.8 [−59.1 to −44.5]	<0.0001	−11.1 [−22.3 to 0]	1.00
Insulin	−39.1 [−48.5 to −29.8]	<0.0001	−35.8 [−45.7 to −25.9]	<0.0001	−19.8 [−38.9 to −0.8]	0.96
GLP-1	−17.6 [−28.7 to −6.5]	0.05	−17.8 [−29.7 to −5.8]	0.09	−10.3 [−33.6 to 13]	1.00
PYY	−15.9 [−27.3 to −4.5]	0.15	−18.1 [−30.4 to −5.8]	0.10	−21.6 [−45.6 to 2.4]	1.00
α-MSH	10.8 [−0.8 to 22.5]	1.00	16.2 [3.9 to 28.5]	0.23	24.2 [0.3 to 48.1]	1.00
Des-acyl ghrelin	29.2 [18.8 to 39.6]	<0.0001	29 [18.2 to 39.8]	<0.0001	14.4 [−6.4 to 35.2]	1.00
Acyl ghrelin	25.7 [14.9 to 36.5]	0.0002	25.7 [14.3 to 37.1]	0.0004	14.6 [−7.4 to 36.7]	1.00

* Interpretable as a difference of percentile means of the considered value; ** *p*-value adjusted by the Bonferroni method on the 23 comparisons (between all biological studied factors); ^†^ adjusted on age (linear effect) and sex; ^‡^ adjusted on age (linear effect), sex, and BMI (linear effect).

**Table 3:** Linear models explaining plasma IgG concentrations of peptides associated with food intake according different adjustments.

	Plasma Anti-Peptide IgG/ Kd	Restrictive vs. Compulsive * Unadjusted Models	*p* **	Restrictive vs Compulsive Adjusted Models 1 ^†^	*p* **	Restrictive vs Compulsive * Adjusted Models 2 ^‡^	*p* **
Plasma anti-peptide IgG	Anti-leptin	−6.5 [−18.2 to 5.2]	1.00	−8.5 [−21.1 to 4.2]	1.00	−17.6 [−42.2 to 7]	1.00
	Anti-insulin	8 [−3.5 to 19.5]	1.00	7.8 [−4.4 to 19.9]	1.00	0.2 [−23.4 to 23.9]	1.00
	Anti-GLP-1	10.1 [−1.3 to 21.5]	1.00	10.1 [−2 to 22.2]	1.00	−0.7 [−24.2 to 22.9]	1.00
	Anti-PYY	5.9 [−5.6 to 17.5]	1.00	3.5 [−8.9 to 15.8]	1.00	−5.9 [−30 to 18.1]	1.00
	Anti-α-MSH	4.3 [−7.3 to 15.9]	1.00	−0.7 [−12.8 to 11.5]	1.00	−1.1 [−24.9 to 22.7]	1.00
	Anti-Acyl ghrelin	4.1 [−7.5 to 15.7]	1.00	−0.2 [−12.5 to 12.2]	1.00	−12.9 [−37 to 11.1]	1.00
	Anti-Des-acyl ghrelin	−9.3 [−21 to 2.3]	1.00	−10 [−22.6 to 2.6]	1.00	−5.2 [−29.7 to 19.3]	1.00
	Anti-ClpB	4.9 [−6.6 to 16.4]	1.00	6.8 [−5.6 to 19.1]	1.00	5.1 [−19 to 29.1]	1.00
Kd of plasma IgG	Anti-leptin	−5.5 [−17.2 to 6.3]	1.00	−6.5 [−19.2 to 6.2]	1.00	−18.3 [−43 to 6.4]	1.00
	Anti-insulin	−6 [−17.7 to 5.8]	1.00	−6.9 [−19.6 to 5.7]	1.00	−11.4 [−36.2 to 13.4]	1.00
	Anti-GLP1	−5.4 [−17.1 to 6.3]	1.00	−4.2 [−16.9 to 8.5]	1.00	−9 [−33.8 to 15.7]	1.00
	Anti-PYY	−5.6 [−17.3 to 6.2]	1.00	−4.9 [−17.5 to 7.8]	1.00	−7.5 [−32.2 to 17.2]	1.00
	Anti-α-MSH	0.5 [−11.2 to 12.3]	1.00	0.9 [−11.7 to 13.5]	1.00	9.3 [−15.2 to 33.9]	1.00
	**Anti-Acyl ghrelin**	**13.9 [2.4 to 25.5]**	**0.42**	**13.1 [0.6 to 25.5]**	**0.92**	16.7 [−7.7 to 41]	1.00
	Anti-des-acyl ghrelin	−3.6 [−15.3 to 8.2]	1.00	−4.7 [−17.3 to 8]	1.00	−6.1 [−30.8 to 18.7]	1.00
	**Anti-ClpB**	**15.8 [4.3 to 27.2]**	**0.17**	**16.4 [4 to 28.8]**	**0.23**	0.2 [−23.7 to 24.1]	1.00

* Interpretable as a difference of percentile means of the considered value; ** *p*-value adjusted by the Bonferroni method on the 23 comparisons (between all biological studied factors); † adjusted on age (linear effect) and sex; ‡ adjusted on age (linear effect), sex, and BMI (linear effect). Statistically significant results are in bold.

## Abbreviations

α-MSH	alpha-Melanocyte-stimulating hormone
AN	Anorexia nervosa
BN	Bulimia nervosa
BED	Binge eating disorder
BSQ	Body Shape Questionnaire
ED	Eating Disorder
EDI-2	Eating Disorder Inventory
HAD	Hospital Anxiety and Depression
IgG	Immunoglobulin G
OSFED	Other Specified Feeding or Eating Disorders
UFED	Unspecified Feeding or Eating Disorders
